# Relationship between Resting State Heart Rate Variability and Sleep Quality in Older Adults with Mild Cognitive Impairment

**DOI:** 10.3390/ijerph182413321

**Published:** 2021-12-17

**Authors:** Bernhard Grässler, Milos Dordevic, Fabian Herold, Sabine Darius, Corinna Langhans, Nicole Halfpaap, Berit K. Labott, Patrick Müller, Achraf Ammar, Beatrice Thielmann, Irina Böckelmann, Notger G. Müller, Anita Hökelmann

**Affiliations:** 1 Department of Sport Science, Faculty of Humanities, Otto von Guericke University, 39106 Magdeburg, Germany; corinna.langhans@ovgu.de (C.L.); nicole.halfpaap@ovgu.de (N.H.); berit.labott@ovgu.de (B.K.L.); achraf1.ammar@ovgu.de (A.A.); anita.hoekelmann@ovgu.de (A.H.); 2 Research Group Neuroprotection, German Center for Neurodegenerative Diseases (DZNE), 39120 Magdeburg, Germany; milos.dordevic@uni-potsdam.de (M.D.); fabian.herold@uni-potsdam.de (F.H.); patrick.mueller@dzne.de (P.M.); notger.mueller@uni-potsdam.de (N.G.M.); 3 Department of Neurology, Medical Faculty, Otto von Guericke University, 39120 Magdeburg, Germany; 4 Research Group Degenerative and Chronic Diseases, Movement, Faculty of Health Sciences, University of Potsdam, 14469 Potsdam, Germany; 5 Department of Occupational Medicine, Medical Faculty, Otto von Guericke University, 39120 Magdeburg, Germany; sabine.darius@med.ovgu.de (S.D.); beatrice.thielmann@med.ovgu.de (B.T.); irina.boeckelmann@med.ovgu.de (I.B.); 6 Center for Behavioral Brain Sciences (CBBS), Brenneckestraße 6, 39118 Magdeburg, Germany

**Keywords:** heart rate variability, cardiac autonomic control, sleep, mild cognitive impairment, cardiovascular health

## Abstract

Sleep problems can be caused by psychological stress but are also related to cardiovascular and neurodegenerative diseases. Improving lifestyle behaviors, such as good sleep hygiene, can help to counteract the negative effects of neurodegenerative diseases and to improve quality of life. The purpose of this cross-sectional study was to investigate the relationship between subjectively reported measures of sleep quality (via Pittsburgh Sleep Quality Index (PSQI)) and objective measures of cardiac autonomic control (via resting state heart rate variability (HRV)) among individuals with mild cognitive impairment (MCI). The PSQI and resting state HRV data of 42 MCI participants (69.0 ± 5.5; 56–80 years) were analyzed. Nineteen of the participants reported poor sleep quality (PSQI score > 5). Good sleepers showed higher resting heart rate than bad sleepers (*p* = 0.037; ES = 0.670). Correlation analysis showed a significant correlation between the parameter HF nu and sleep efficiency, contrasting the expected positive association between reduced HRV and poor sleep quality in healthy and individuals with specific diseases. Otherwise, there were no significances, indicating that measures of subjective sleep quality and resting HRV were not related in the present sample of MCI participants. Further research is needed to better understand the complex relationship between HRV and lifestyle factors (e.g., sleep) in MCI.

## 1. Introduction

Worldwide, the proportion of older adults is increasing, leading to a decreased proportion of younger relative to older age groups. The proportion of people over 65 years is expected to rise from 9.3% in 2020 to 22.6% in 2100 [1]. This development implies new challenges for the health care system due to the parallel increase in age-related diseases, such as neurodegenerative diseases [2]. Accordingly, the number of individuals with MCI (mild cognitive impairments) increases. MCI is characterized by mild cognitive changes (i) that are serious enough to be noticed by the person affected and by their family members and (ii) that can be evaluated by using objective measures that typically reveal impairments in cognitive domains such as memory, attention, or executive functioning [3]. In the stage of MCI, the cognitive deficits do not significantly affect activities of daily living [3]. Moreover, it can be distinguished between two subtypes of MCI, namely amnestic MCI (with memory impairments) or non-amnestic MCI (without memory impairments) [4]. The incidence of MCI among individuals aged 75 to 79 years is about 22.5 per 1000 persons and increases to 60.1 for individuals aged 80 to 84 years [5]. A meta-analysis showed that the annual conversion rate is about 7% from MCI to dementia and Alzheimer’s disease (AD) [6]. AD is one of the most common neurodegenerative diseases in later life. Each year, approximately 10 million older individuals worldwide are diagnosed with AD [2]. As there are currently no effective treatments to cure AD [7], a better understanding of the pathological processes associated with this disease and an early detection of AD are essential to initiate appropriate interventions [8]. Since individuals with MCI are at risk of developing dementia later in their life, the construct of MCI allows for a timely onset of interventions aiming to deaccelerate cognitive decline [9].

Lifestyle factors (including diet, physical activity, alcohol, and nicotine consumption) can influence the progression of MCI [3,10,11,12,13]. Among these lifestyle factors, sleep is one modifiable risk factor [14,15] that can accelerate or deaccelerate cognitive decline [15,16,17]. Sleep is a complex bio-physiological process controlled by the basal forebrain [14] and related to mental and physical health [18]. Studies also showed poor sleep quality in individuals with MCI [19,20,21,22,23]. For example, two recent reviews have demonstrated a positive association between cognitive decline and poor sleep quality [24,25]. Poor sleep favors cognitive decline and disease progression [26,27,28,29] by affecting, for instance, hippocampal volume [30,31]. In addition, the decline in sleep quality is related to cell loss in the forebrain [32,33]. Studies have shown that people with AD and MCI have a reduced sleep quality compared to healthy people, as measured by objective methods such as polysomnography (PSG) [34]. In a longitudinal study, it was found that the decline in cognitive function is related to an impaired sleep quality and problems in falling asleep [35]. Shortness of sleep is likely to increase the risk of cognitive impairment, as it promotes inflammatory processes in the human organism [36,37]. In turn, other studies have shown that very long sleep duration is also related to an increased risk of MCI [15,38,39,40]. Therefore, a U-shaped relationship between sleep duration and cognitive impairment seems plausible [41]. In summary, several studies reported a relationship between sleep disturbances and cognitive dysfunction [34,35,42] with one longitudinal study reporting an association between poor sleep quality and increased MCI risk [35].

The autonomic nervous system (ANS) plays an important role in the regulation of sleep by controlling cardiovascular function and the transition between sleep phases [43]. One parameter assessing the functioning of the ANS is heart rate variability (HRV). HRV reflects the cardiac autonomic control and is considered as a measure of neurocardiac function [44,45]. It is generated by the complex interaction between the sympathetic and parasympathetic nervous systems [46] and regulatory mechanisms controlling heart rate [45]. The latter includes the baroreceptor reflex [47] and rhythmic changes in vascular tone [45]. Reduced HRV indicates impairment of the cardiovascular system and brain–heart interaction, a dominance of the sympathetic over the parasympathetic nervous system, indicating a high state of stress, which has an unfavorable impact on health status [46,48,49]. Conversely, relatively high HRV characterizes a healthy organism and functioning of cardiac autonomic control as well as high adaptive capacity and resilience [44,50].

HRV can be quantified using time-domain, frequency-domain, and non-linear measures [51,52]. The most commonly used time-domain parameters include SDNN (standard deviation of the NN intervals) and RMSSD (square root of the mean squared differences of successive NN intervals). The most commonly used frequency-domain parameters are power in the high-frequency range (HF; 0.15–0.4 Hz) and low-frequency range (LF; 0.04–0.15 Hz). Both measures can also be expressed in relation to total power in normalized units. RMSSD and HF are the primary indices reflecting parasympathetic control. Thus, these indices are commonly used in psychophysiological research [53]. Non-linear parameters have been rarely used but are becoming more popular as they better represent heart rate complexity [54]. One common non-linear parameter is D2 (correlation dimension) reflecting self-similarity of NN intervals [51,52,54]. HRV as a measure of cardiac autonomic balance is also associated with cognitive functioning [48,49] with a positive correlation between a relatively high HRV and better cognitive functioning [55,56]. This relationship can be explained by the neurovisceral integration model [57].

HRV has also been widely used to assess sleep quality. Several prior studies had suggested a positive relationship between HRV and sleep quality [18,58,59,60]. For example, in the study of Hsu et al. [18], sleep quality and the resting state HRV (from 5 min recordings) were positively correlated in their sample of female nurses, indicating an association between lower sleep quality and higher sympathetic and lower parasympathetic activity [61]. A correlation between HRV and sleep disturbances was also observed in patients with panic disorder [62]. Comparable to the findings of Hovland et al. [62] and Hsu et al. [18], a positive relationship between sleep quality and short-term HRV in healthy adults was observed in a recent study [60]. This finding indicates an autonomic imbalance with sympathetic dominance over parasympathetic control in adults with sleep problems [61].

In a study with healthy adults, participants who sleep less than six hours had a higher resting heart rate compared to participants who sleep more than seven hours [63]. Furthermore, the HRV of participants who sleep less or had a lower sleep efficiency was lower than in participants who sleep more than seven hours. Interestingly, sleep duration was not related to short-term measures of resting state HRV [64] nor nighttime HRV [65] in healthy adults. Therefore, the extent to which very short or very long sleep duration affects cardiac autonomic control remains to be elucidated.

There is mixed evidence regarding the relationship between 24 h, daytime, and nighttime HRV and sleep quality. A study investigating the relationship between HRV and sleep quality in 199 healthy women showed no correlations for nighttime but for daytime HRV [61]. No correlation between sleep quality and nighttime HRV in 84 healthy, middle-aged men and women was found in the study of Schierholz [66]. Other studies [61,66,67,68] proposed that nighttime HRV and sleep quality are not associated, since different sleep stages affect the heart rate as well as the sympathetic and parasympathetic activity differently. In contrast, Burton et al. [67] found a significant relationship between reduced sleep quality and lower RMSSD and heart rate during sleep. Regarding 24 h HRV, significant negative correlations were reported between sleep quality and HRV [66,69] and positive correlations were reported between sleep quality and mean heart rate [69]. The investigation of Yang et al. [70] showed that the relationship between sleep quality and HRV is not consistent in different groups of participants, as they observed correlations between sleep quality and HRV only in patients with depression but not in healthy participants and insomniacs.

We are not aware of any study to date that has examined the relationship between sleep quality and HRV in individuals with MCI. In order to develop effective treatments, a better understanding of the relationship between MCI and lifestyle factors such as sleep is important. Given that cardiovascular and ANS diseases are related to cognitive decline [63], assessing cardiac autonomic control through HRV is a common approach. The present study aims to investigate the association between subjective measures of sleep quality (via PSQI) and cardiac autonomic control (via HRV at resting state) in older adults with MCI to determine whether reduced HRV and poor sleep quality are related. Based on the findings of previous studies [18,58,60], we hypothesize that a positive relationship between subjective measures of sleep quality and resting state HRV in individuals with MCI exist. We also aimed to assess whether HRV and sleep quality differed between good and bad sleepers as well as between individuals with amnestic MCI (aMCI) and non-amnestic MCI (naMCI).

## 2. Materials and Methods

### 2.1. Participants

Through advertisements in local newspapers, flyers, posters, word of mouth, and using existing databases, 219 adults were initially recruited. After recruitment, participants were screened for eligibility based on the following inclusion criteria: 50 to 80 years of age; native German-speaking; and living and able to manage everyday activities independently. Exclusion criteria were as follows: neurological diseases other than MCI (i.e., epilepsy, multiple sclerosis); severe known cardiac diseases, as these could influence HRV [71] (i.e., severe cardiac insufficiency, cardiac pacemaker, valvular defect, arterial hypertension, cardiac arrhythmias, atrial fibrillation); mental diseases (i.e., schizophrenia, depression (score > 5 in the Geriatric Depression Scale (GDS) [72]); orthopedic diseases (i.e., bone fracture in last six months, symptomatic slipped disc); muscular diseases (i.e., myositis, tendovaginitis); severe endocrinologic diseases (i.e., manifest hypothyroidism or hyperthyroidism, insulin-dependent diabetes mellitus type II); injury or surgery in the last six months; consumption of illegal intoxicants or alcohol abuse; uncorrected poor eyesight or hearing; anamnestic known color blindness or red–green weakness; pregnancy or breastfeeding; using neuroleptics, narcotic analgesics, benzodiazepines, or psychoactive medications. Further, eligible participants were administered the CERAD (Consortium to Establish a Registry for Alzheimer’s Disease) plus test battery to detect the presence of cognitive impairment [73]. Participants who scored 1.5 z-scores below the age- and education-adjusted reference sample in at least one subtest of the CERAD plus test battery were referred to experienced neurologists for further diagnosis. MCI diagnosis was made in accordance to the guidelines of Petersen et al. [74]. To differentiate between aMCI and naMCI, we used the subtests Wordlist and Figure Saving entailed in the CERAD test battery. As recommended by Jessen et al. [73], participants with a performance below −1.5 SD in the age, sex, and education-adjusted z-score in one of these subtests were classified as aMCI. The workup included taking the medical history, assessment of daily activities, checking of the evaluation of depressive symptoms (via GDS), neurological examination, and laboratory blood tests (e.g., brain-derived neurotrophic factor). In addition, every participant received an MRI to exclude severe brain pathology (e.g., vascular encephalopathy).

**Figure 1:**
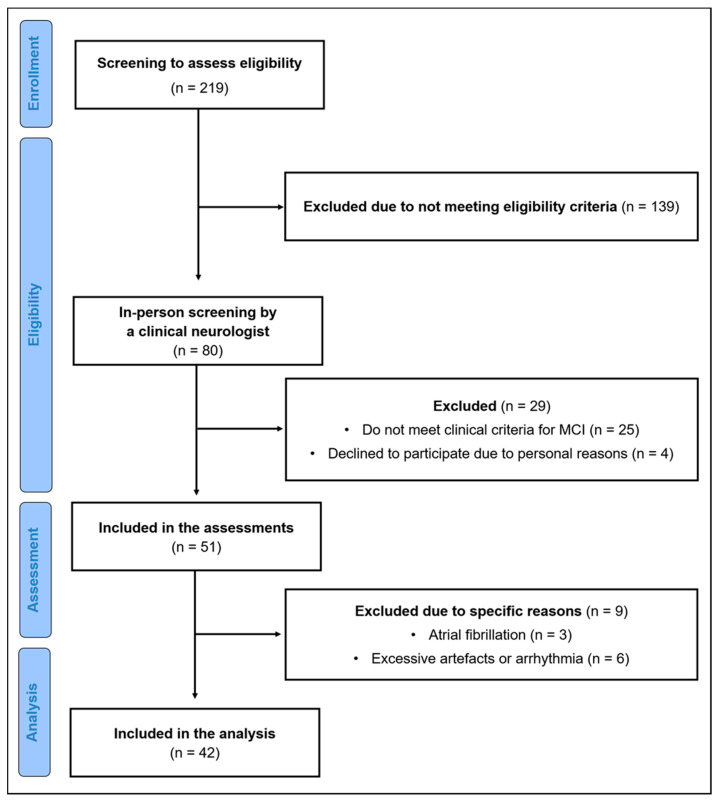
Flow diagram of participants.

After exclusion of participants that did not meet our criteria, 51 participants were included for assessment. Nine participants were excluded from the analysis due to excessive artefacts (>5%) in the Electrocardiogram (ECG) data, arrhythmia (*n* = 6) or atrial fibrillation (*n* = 3), resulting in 42 participants for the final analysis. A flow diagram of the sample selection process is shown in Figure 1.

### 2.2. Ethical Approval

This study was approved by the Ethics Committee of the Otto von Guericke University Magdeburg (reference number: 83/19), and the study procedures are in accordance with the latest version of the Declaration of Helsinki. The study was pre-registered in ClinicalTrials.gov (NCT04427436) on the 10 June 2020.

### 2.3. Experimental Design

All measurements were conducted in laboratories at the Otto von Guericke University Magdeburg. Prior to the assessment, all participants were briefed about the experimental procedure and informed of possible risks and benefits associated with the study. Thereafter, participants provided written consent to participate and received financial compensation after completing the test battery.

Participants were instructed to refrain from intense physical training and drinking alcohol 24 h before the HRV recording. Drinking caffeinated drinks, smoking, and eating were not allowed two hours before the experiment to limit the potential acute effects on HRV. Participants were also requested to follow their normal sleep behavior before the day of the measurement. ECG data were collected at resting state according to recent recommendations for psychophysiological research [53]. A more detailed description of the measurement procedures can be found in the previously published study protocol [75].

### 2.4. Subjective Sleep Quality

Subjective sleep quality was assessed using the German version [76] of the PSQI (Pittsburgh Sleep Quality Index) [77]. The PSQI is a self-rated questionnaire assessing sleep quality and disturbances over the past four weeks. It comprises 18 items within seven components: subjective sleep quality, sleep latency, sleep duration, habitual sleep efficiency, sleep disturbances, use of sleeping medication, and daytime dysfunction. Participants were instructed to answer each item on a scale from 0 to 3. The scores of each component are summed up to the global score of subjective sleep quality (0–21). Higher scores indicate poor subjective sleep quality. A score above 5 was used as cut-off to differentiate between good sleepers and bad sleepers with a sensitivity of 89.6% and a specificity of 86.5% [77]. The internal consistency is indicated by a Cronbach’s alpha coefficient of 0.83 [77]. The questionnaire was handed out after the MCI diagnosis. There was a maximum of two weeks between the HRV measurement and the completion of the questionnaire.

### 2.5. Autonomic Data Collection and Processing

HRV measurements were conducted according to recent recommendations [53]. Participants were sitting on a comfortable chair, knees were bent at a 90° angle, hands on their thighs, advised to relax, and breath normally. To avoid artefacts, participants were instructed not to move or talk during the recording. The resting state measurement lasted for five minutes with a stabilization period of five minutes prior to the measurement to ensure a relaxed state.

Electrocardiographic data were collected with a three-channel Holter-ECG with a sampling rate of 1000 Hz (Medilog AR12plus, Schiller Medizintechnik GmbH, Baar, Switzerland). The raw ECG data were uploaded to the Medilog Darwin 2 analysis software package (Schiller Medizintechnik GmbH, Baar, Switzerland) and then checked automatically and visually by a healthcare professional for clinical abnormalities. Afterwards, text files comprising consecutive NN intervals were generated. Finally, HRV analysis was performed using the Kubios premium 3.3 software package (University of Kuopio, Kuopio, Finland). Artefact correction was done with an artefact identification threshold of 0.3 s and a smoothness priors method for detrending NN intervals (Lambda = 500, fc = 0.035 Hz) according to the national guideline and literature [51,52].

HRV analysis comprised time- and frequency-domain and non-linear parameters. The time-domain index was RMSSD (ms). Spectral analysis for the frequency-domain index, HF nu, was performed by Autoregression using model order 16, as recommended by Laborde et al. [53]. As non-linear measures have been rarely used in the evaluation of cardiac autonomic control, the non-linear index D2 was used as the parameter reflecting heart rate complexity [54]. Higher values indicate greater complexity [45] and adaptability of the cardiac system, whereas lower values indicate a shift toward sympathetic dominance [54]. In addition to HRV parameters, the mean heart rate (mHR) was also assessed.

### 2.6. Statistical Analysis

Data were analyzed using SPSS version 26.0 (SPSS Inc., Chicago, Il, USA) on Windows version 10. Shapiro–Wilk’s test was used to check for the normality of all data. In order to compare the differences between good and bad sleepers, and aMCI and naMCI, *t*-tests and Mann–Whitney *U*-tests were used. Effect size d was reported for *t*-tests indicating small (d > 0.2), medium (d > 0.5), and large (d > 0.8) effects, and effect size r was reported for *U*-tests indicating small (>0.1), medium (>0.3), and large (>0.5) effects [78]. Non-parametric Spearman’s partial correlation was applied, controlling for age and sex, to quantify the relationship between HRV parameters and PSQI components, i.e., subjective sleep quality, sleep latency, sleep duration, habitual sleep efficiency, sleep disturbances, use of sleeping medication, and day-time dysfunction, as well as mean global score [79]. *p* < 0.05 was considered statistically significant and two-sided. We reported the effect size rho and rated effects as small (>0.1), medium (>0.3), and large (>0.5) [78].

## 3. Results

### 3.1. Characteristics of the Study Sample

Table 1 presents the demographic characteristics and HRV data of the study sample. In total, 42 (19 men, 23 women) participants were included in the final statistical analysis. The age ranged from 56 to 80 years. The years of education ranged from 11 to 20 years. The Mini Mental State Examination (MMSE) score ranged from 25 to 30. Twenty-three participants were classified as good sleepers (PSQI score ≤ 5) and 19 participants were classified as bad sleepers (PSQI score > 5). Both groups were similar in terms of age, sex distribution, body composition (height, weight, and BMI), years of education, MMSE, RMSSD, HF nu, and D2. Good sleepers showed significantly higher mean heart rate (mHR) (*p* = 0.037; d = 0.670). The general characteristics and HRV data of naMCI and aMCI participants are shown in Supplementary Table S1.

### 3.2. Subjective Sleep Quality

The results of the PSQI components and mean global scores are shown in Table 2. The mean global PSQI score of the total study sample was 5.55 ± 3.15. Bad and good sleepers differed significantly in all components. Bad sleepers showed higher scores in global score, sleep latency, sleep duration, sleep efficiency, and sleep disturbance with *p* ≤ 0.001 and r = 0.526 to 0.803; subjective sleep quality and sleep medication with *p* ≤ 0.01 and r = 0.482 and 0.395, respectively; and sleep dysfunction (*p* = 0.036; r = 0.345). PSQI scores of both MCI groups and group differences are shown in Supplementary Table S2. No significant differences were observed.

### 3.3. Correlation between HRV and PSQI Components

Table 3 presents the results of the partial correlation analysis between HRV parameters and PSQI scores. There was one significant positive correlation between HF nu and sleep efficiency (*p* = 0.048; r = 0.314).

## 4. Discussion

The present study aimed to investigate the relationship between subjective measures of sleep quality and resting state HRV among older adults with MCI. Nineteen of 42 participants had a global score in the PSQI of >5 indicating poor sleep quality and thus were considered as bad sleepers. Good sleepers had significantly higher mHR, but both groups differed neither in HRV nor demographic data. There was no difference in HRV nor sleep quality between aMCI and naMCI. Furthermore, only one significant correlation between resting state HRV and subjective measures of sleep quality was observed, namely between HF nu and sleep efficiency, indicating lower efficiency with higher short-term HRV. The results of the correlation analysis do not support our hypothesis of a positive relationship between subjective measures of sleep quality and resting state HRV in older individuals with MCI.

There is evidence in the literature that sleep problems and a decline in sleep quality are related to the incidence of dementia, suggesting that sleep problems might constitute an early warning sign of imminent cognitive decline [40,80]. For instance, 70% of patients in an early stage of dementia reported sleep problems [68]. Although the causal mechanisms between sleep and brain integrity have not been fully understood, sleep problems can negatively influence the brain structure given that sleep disturbances have been linked to a more pronounced cortical atrophy [81,82,83]. Additionally, shorter sleep duration can adversely affect neuronal excitability, synaptic plasticity, and neurogenesis that, in turn, precipitate hippocampal degeneration [84,85].

The lack of a significant correlation between resting state HRV and sleep quality in the present study contradicts previous research reporting associations between sleep quality and HRV in healthy individuals [18,58,67], in patients with chronic fatigue syndrome [67], depression [70] or panic disorder [62]. These divergent results may have several reasons. First, a short-term measurement of HRV provides only limited insights into the current state of cardiac autonomic control, as it is more or less a “snapshot” of the actual state of the ANS. Therefore, the associations between PSQI score and HRV may have been biased. An alternative approach to gain more insights into the complex relationship between HRV and measures of sleep could be to quantify HRV over the course of 24 h and thus also during sleep [61,67,70]. Furthermore, the results showed that the HRV and subjective sleep quality of the sample was very homogeneous; with that lack of variance, smaller associations were not detectable. Finally, while the PSQI is a well-established questionnaire [86,87], the self-reports might be not as accurate as or tap into dimensions other than objective measures of sleep (e.g., derived by PSG or actigraphy). Thus, our findings should be interpreted in the light of this limitation. In addition, although the PSQI has been previously used in older adults with MCI [88,89,90], the reliability of subjective measures of sleep quality has, to the best of our knowledge, not been firmly established in this particular cohort and might be a function of the severity of cognitive deficits. However, in older adults being at higher risk of dementia, the retest reliability of subscales ranges between fair and excellent [91] but is excellent for global PSQI score [92]. Nevertheless, future studies should also use objective assessment tools (e.g., PSG or actigraphy) in addition to PSQI to assess individual sleep patterns more comprehensively.

The mean PSQI global score of the MCI participants in the present study was 5.55. This is similar to other studies examining sleep quality and HRV in younger healthy participants [60,66]. In these studies, the mean PSQI global scores were 5.5 and 6.6, respectively. A slightly lower value of 4.1 was found in the study of Werner et al. [65]. However, the mean age of these participants was 23.6 years, which was much lower than in our study with a mean age of 69.0 years. The score for the subcomponent sleep quality in our study was 1.50, which is similar to the score of 1.12 in another study with younger participants [66]. The mean PSQI global score in a study with AD and MCI patients was 5.7 and 6.2, respectively [42], which is also similar to the present study. However, the score of the healthy control group was 4.9 and thus slightly lower than that of the AD or MCI group [42]. Furthermore, the subcomponent sleep quality was 0.8, 0.9., and 0.8 in AD, MCI, and healthy participants, and therefore slightly lower than in the present study. Other groups of participants showed much poor PSQI scores. In female nurses, the global score was 10.2 [18], in depressed patients, it was 12.8, and in insomniacs, it was 10.7 [70]. Patients with panic disorder had a global score of 8.0 [62]. The subcomponent of subjective sleep quality was also much higher in depressive patients and insomniacs (2.4) than in MCI participants of our and another study [42].

Collectively, daily psychological stress can affect the subjective sleep quality, but the available data regarding sleep quality in MCI participants, as assessed by the PSQI, is relatively limited at the moment. It is likely that the PSQI is not sensitive enough to observe subtle differences between MCI and healthy controls, buttressing the need to combine it with more objective measures of sleep quality. Previous studies presented only the mean global score but not the scores of the subcomponents of the PSQI [18,60,62,65]. For a better comparison across studies, future research in this field is encouraged to present both the mean global score and the subcomponent scores of the PSQI. To date, there is only one longitudinal study available investigating the incidence of MCI and the change in sleep quality [35]. In this study, a decrease in sleep quality was related to the incidence of MCI. However, no biomarkers had been collected, and thus, no additional information on the underlying pathologies for the occurrence of MCI was provided. Moreover, the MCI diagnosis was not based on an objective clinical procedure, limiting the overall conclusion that can be drawn.

A few limitations of the study have to be mentioned. Firstly, there was a certain time delay between HRV recording and assessment of subjective sleep quality via PSQI. Given that short-term HRV is only a reflection of the actual state of cardiac autonomic control, it is influenced by chronic changes (e.g., sleep patterns over the last four weeks as assessed by PSQI) but also by acute changes (e.g., sleep patterns in the last night). Thus, acute changes in sleep patterns might act as possible confounder and should be considered in future studies. In addition, a long-term 24 h recording could provide more accurate insight as it allows assessing nighttime HRV. However, 24 h recordings are more stressful for the participants. Furthermore, the small sample size might have prevented significant correlations. Therefore, larger samples should be tested in future studies to increase statistical power. Finally, we tested a high functioning sample of MCI patients with rather high MMSE scores. Although this should not compromise the relationship between HRV and sleep quality, comparison of the absolute HRV and PSQI values with previous studies is difficult.

## 5. Conclusions

The findings of the present study suggested that subjectively reported sleep quality was not related to resting state cardiac autonomic control in participants with MCI. Furthermore, we observed that older individuals with aMCI and naMCI did not differ concerning their HRV and sleep quality. Based on our findings, we recommend that future studies (i) should include patients with manifest AD to investigate how sleep quality and HRV change in the continuum of cognitive health (e.g., healthy older adults vs. MCI vs. AD), (ii) should consider subjective (e.g., PSQI) and objective measures of sleep quality (e.g., PSG or actigraphy), (iii) should investigate longitudinal changes in HRV, sleep quality, and cognitive performance in order to rule out possible relationships between those measures, and (iv) should consider lifestyle factors other than sleep, such as diet, stress, and physical activity, to assess the complex relationships between cognitive performance, measures of sleep, and HRV more comprehensively.

## Figures and Tables

**Table 1:** Characteristics of participants and HRV data (Mean ± SD).

Variables	Total (*n* = 42)	Bad Sleepers (*n* = 19)	Good Sleepers (*n* = 23)	*p*-Value	Effect Size
Age (years)	69.0 ± 5.5	69.3 ± 4.8	68.8 ± 6.1	0.711 ^a^	0.090
Male/female (*n*)	19/23	8/11	11/12	0.606 ^b^	0.114
Height (cm)	170.69 ± 8.81	170.42 ± 9.22	170.91 ± 8.67	0.860 ^a^	0. 055
Body weight (kg)	74.04 ± 11.58	72.32 ± 10.01	75.47 ± 12.77	0.595 ^c^	0.134
BMI (kg/m^2^)	25.32 ± 2.53	24.83 ± 2.10	25.72 ± 2.81	0.259 ^a^	0.354
Years of education (years)	15.33 ± 2.52	14.95 ± 2.00	15.65 ± 2.89	0.373 ^a^	0.277
MMSE (score)	27.24 ± 1.23	26.84 ± 0.83	27.57 ± 1.41	0.092 ^c^	0.294
mHR (bpm)	67.09 ± 9.89	63.61 ± 8.84	69.96 ± 9.96	0.037 ^a,^*	0.670
RMSSD (ms)	25.97 ± 15.76	30.16 ± 15.72	22.52 ± 15.27	0.063 ^c^	0.240
HF nu	47.82 ± 21.25	54.80 ± 22.51	42.05 ± 18.71	0.093 ^c^	0.297
D2	0.75 ± 1.32	0.96 ± 1.49	0.58 ± 1.16	0.519 ^c^	0.143

HRV: heart rate variability; BMI: body mass index; MMSE: Mini Mental State Examination; mHR: mean heart rate; RMSSD: square root of the mean squared differences of successive NN intervals; HF nu: high frequency power in normalized units. ^a^ Student’s *t*-test; ^b^ Chi-squared test; ^c^ Mann–Whitney *U*-test. * *p* < 0.05.

**Table 2:** Mean global and component PSQI scores (mean ± SD).

Variables	Total (*n* = 42)	Bad Sleeper (*n* = 19)	Good Sleepers (*n* = 23)	*p*-Value	Effect Size
Subjective sleep quality	1.50 ± 0.74	1.89 ± 0.66	1.17 ± 0.65	0.002 **	0.482
Sleep latency	0.86 ± 0.98	1.53 ± 0.96	0.30 ± 0.56	<0.001 ***	0.626
Sleep duration	0.45 ± 0.80	0.89 ± 0.94	0.09 ± 0.42	0.001 **	0.494
Sleep efficiency	0.50 ± 0.86	1.00 ± 1.05	0.09 ± 0.29	<0.001 ***	0.526
Sleep disturbance	1.33 ± 0.53	1.63 ± 0.50	1.09 ± 0.42	0.001 **	0.508
Sleep medication	0.12 ± 0.33	0.26 ± 0.45	0.00 ± 0.00	0.010 *	0.395
Sleep dysfunction	0.81 ± 0.77	1.11 ± 0.88	0.57 ± 0.59	0.036 *	0.345
Global score	5.55 ± 3.15	8.32 ± 2.43	3.26 ± 1.25	<0.001 ***	0.803

PSQI: Pittsburgh Sleep Quality Index; SD: standard deviation. Group differences were based on Mann–Whitney *U*-test. * *p* < 0.05; ** *p* < 0.01; *** *p* < 0.001.

**Table 3:** Non-parametric Spearman’s partial correlation analysis between HRV and PSQI scores (*n* = 42).

Variables	mHR		RMSSD		HF nu		D2	
	*p*-Value	rho	*p*-Value	rho	*p*-Value	rho	*p*-Value	rho
Sleep quality	0.174	−0.219	0.390	0.140	0.344	0.154	0.766	−0.049
Latency	0.512	−0.107	0.987	0.003	0.054	0.307	0.279	−0.176
Duration	0.106	−0.259	0.135	0.240	0.548	0.098	0.725	0.057
Efficiency	0.156	−0.229	0.099	0.264	0.048 *	0.314	0.948	−0.011
Disturbance	0.590	−0.088	0.927	−0.015	0.600	−0.086	0.490	0.112
Medication	0.522	0.104	0.781	−0.045	0.560	0.095	0.920	−0.016
Dysfunction	0.666	−0.070	0.653	0.073	0.579	−0.090	0.448	0.123
Global score	0.132	−0.239	0.251	0.184	0.177	0.215	0.989	0.002

HRV: heart rate variability; PSQI: Pittsburgh Sleep Quality Index; mHR: mean heart rate; RMSSD: square root of the mean squared differences of successive NN intervals; HF nu: high-frequency power in normalized units. * *p* < 0.05.

## Data Availability

Data are available from the corresponding author upon reasonable request.

## Supplementary Materials

The following are available online at https://www.mdpi.com/article/10.3390/ijerph182413321/s1, Table S1: Characteristics and HRV data of naMCI and aMCI individuals (mean ± SD), Table S2: Mean global and component PSQI scores of naMCI and aMCI individuals (mean ± SD).
