# Supplementary material for: Relationship between Resting State Heart Rate Variability and Sleep Quality in Older Adults with Mild Cognitive Impairment
Source: Int J Environ Res Public Health. 2021 Dec 17;18(24):13321. doi: 10.3390/ijerph182413321 (PMC8703743; doi:10.3390/ijerph182413321)
Supplement: Supplementary file 1 [file ijerph-18-13321-s001.zip › ijerph-1446662-SI.pdf]

Table S1. Characteristics and HRV data of naMCI and aMCI individuals (Mean ± SD).

Variables	naMCI (n = 25)	aMCI (n = 18)	p-value	Effect size
Age (years)	69.8 ± 5.8	67.9 ± 4.9	0.295[a]	0.348
Male/female (n)	13/12	6/11	0.286[b]	0.334
Height (cm)	171.36 ± 7.77	169.71 ± 10.34	0.557[a]	0.186
Bodyweight (kg)	72.42 ± 8.68	73.48 ± 15.16	0.336[c]	0.048
BMI (kg/m^2)	25.28 ± 1.73	25.36 ± 3.44	0.931[a]	0.031
Years of education (years)	15.20 ± 2.27	15.39 ± 2.90	0.682[a]	0.075
MMSE (score)	27.24 ± 1.13	27.24 ± 1.39	0.968[c]	0.000
mHR (bpm)	66.24 ± 10.55	68.33 ± 8.99	0.507[a]	0.210
RMSSD (ms)	23.58 ± 15.88	29.49 ± 15.37	0.170[c]	0.185
HF nu	47.47 ± 21.95	48.33 ± 20.82	0.899[a]	0.040
D2	0.43 ± 0.90	1.23 ± 1.68	0.405[c]	0.300

naMCI: nonamnestic mild cognitive impairments; aMCI: amnestic mild cognitive impairments; SD: standard deviation; HRV: heart rate variability; BMI: body mass index; MMSE: Mini Mental State Examination; mHR: mean heart rate; RMSSD: square root of the mean squared differences of successive NN intervals; HF nu: high frequency power in normalized units.

a Student`s t-test; b Chi-squared test; c Mann-Whitney U-test.

Table S2. Mean global and component PSQI scores of naMCI and aMCI individuals (Mean ± SD).

Variables	naMCI (n = 25)	aMCI (n = 18)	p-value	Effect size
Subjective sleep quality	1.64 ± 0.76	1.29 ± 0.69	0.180	0.233
Sleep latency	1.04 ± 0.98	0.59 ± 0.94	0.099	0.227
Sleep duration	0.36 ± 0.76	0.59 ± 0.87	0.309	0.141
Sleep efficiency	0.44 ± 0.87	0.59 ± 0.87	0.405	0.086
Sleep disturbance	1.44 ± 0.58	1.18 ± 0.39	0.085	0.249
Sleep medication	0.16 ± 0.37	0.06 ± 0.24	0.326	0.152
Sleep dysfunction	0.88 ± 0.78	0.71 ± 0.77	0.470	0.109
Global score	5.96 ± 3.34	4.94 ± 2.84	0.401	0.160

PSQI: Pittsburgh Sleep Quality Index; naMCI: nonamnestic mild cognitive impairments; aMCI: amnestic mild cognitive impairments; SD: standard deviation.

Group differences were based on Mann-Whitney U-test.
